# Fairness and Contractual Performance in Vertical Linkages Within an Uncertain Environment: A Case of a Tomato Value Chain

**DOI:** 10.3390/foods13233819

**Published:** 2024-11-27

**Authors:** Amine M. Benmehaia, Antonella Samoggia, Oualid Benharrat, Salah Eddine Benziouche, Georgia Ayfantopoulou

**Affiliations:** 1 Department of Agricultural Sciences, University of Biskra, BP 145 RP, Biskra 07000, Algeria; ma.benmehaia@univ-biskra.dz (A.M.B.); s.benziouche@univ-biskra.dz (S.E.B.); 2 Department of Agricultural and Food Sciences, University of Bologna, Via Zamboni, 33, 40126 Bologna, Italy; 3 Ministry of Agriculture and Rural Development, Algiers 16000, Algeria; ou.benharrat@gmail.com; 4 Hellenic Institute of Transport, Centre for Research and Technology Hellas, P.O. Box 60361, GR 57001 Thessaloniki, Greece; gea@certh.gr

**Keywords:** contract farming, contractual performance, vertical relationship, fair pricing, processed tomatoes, Algeria

## Abstract

In developing countries with uncertain institutional environments, ensuring fairness in contractual arrangements within food value chains is crucial to overcome modern challenges. This paper empirically investigates the vertical relationship between tomato growers and canneries in Algerian agriculture to assess the determinants of contractual performances and price fairness implications. The study is based on an analysis of a comprehensive dataset of 9127 tomato growers engaged in contract farming over four years (2018–2021). Three regression methods were estimated, namely logit, tobit, and quantile regressions, alongside exploratory analysis. The main findings shed light on the factors influencing contractual performance among contracting producers, primarily farm scale and distance from the contracting processor. Additionally, the findings highlight the key role of contract enforcement mechanisms in influencing the contractual performances of tomato growers. Several recommendations are made to incentivize tomato growers and improve overall contractual performance within such public policy settings. Contract arrangements, including fair price negotiation and the requirement for tomato processing firms to make specific investments, are advocated to foster self-enforcement and significantly enhance the growers’ contractual performance. This is particularly important in many developing countries where the business environment is characterized by an absence of effective public enforcement institutions along with a highly uncertain environment.

## 1. Introduction

In food supply chain research, there is a growing body of scientific literature focusing on fairness aspects. Scholars have intensely analyzed actors’ perceptions of fair practices and risks [1,2,3,4,5]. Over the past two decades, food supply chains in developing countries have undergone profound changes in their structure and performance. One significant change is the introduction of contract farming, which has notably affected industry configurations. Evidence suggests an increasing trend of farmer participation in contract farming, particularly in developing countries [6,7,8,9]. This trend is attributed to the benefits of such practices, including improved profits, income stability, and enhanced food safety [10,11,12,13,14]. Enabling close actors to contract into increasingly tightly linked value chains in an uncertain institutional environment affects industrial dynamics and the logic of public intervention. Therefore, rethinking the fairness and dynamics of contractual performances in agro-industrial complexes is a fruitful inquiry to draw reliable conclusions about the future of food supply chains in developing countries.

The complexity of agro-industrial complexes inevitably gives rise to diverse management practices, leading to the emergence of various hybrid forms in food sectors worldwide [15,16]. Recent theoretical frameworks underscore the use of multiple vertical coordination devices to manage these complexities. Vertical coordination, as described by [17,18], refers to the synchronization of successive stages of production and marketing concerning the quantity, quality, and timing of product flows. This coordination is facilitated by region- and sector-specific governance structures, such as formal contracts. The processing industry expects suppliers to offer the quality and quantity of produce needed, usually employing mixed governance forms, including markets, contracts, and some degree of vertical integration [19]. Formal contracts play a key role in the governance of buyer–supplier relationships and often complement relational mechanisms [20,21,22]. The main challenge in contracting practices in food supply chains is contract enforcement; its absence would affect the overall value chain. Depending on the context and the transaction costs involved, contracts can be enforced through various public and private mechanisms, either separately or in combination [23]. As the transaction cost approach was introduced to elicit vertical coordination via contracts in agrifood markets [24,25], contracts function as sophisticated coordination devices to handle complex arrangements for horizontal and vertical coordination [26] in the agrifood complex. Therefore, enforcement issues, regardless of the form, are highly contingent on the actors’ perception of fairness in food supply chains, which are greatly influenced by market vicissitudes.

In developing countries, agricultural products produced under contracts have increased rapidly, acting as a driving force for swift agro-industrialization. In such environments, agricultural production contracts have attracted the interest of practitioners (farmers and processors), theorists, and policymakers [27]. However, this surge in agricultural production contracts has raised various concerns, with one prominent issue being the potential for certain actors along the value chain to exploit prevailing uncertainties and manipulate outcomes. Contract farming has faced criticism as a tool used by agribusiness firms and food multinationals to exploit unequal power relationships with farmers [18,28]. It is often overlooked that the farmer is not the only loser in these dealings. Due to the absence or weakness of enforcement mechanisms, farmers’ judgment of fairness in such dealings is affected. Farmers often find that contracts are biased, with firms (mostly large-scale agribusiness firms) underpricing their services and consistently transferring market risks onto them. The consequences of such a situation in a developing country are significant, raising important questions for policymakers concerned about food security issues.

In many developing contexts, including Algeria, contract farming is a relatively new practice that has intensified under the pressure of industrial development relaunch. While small-scale farming and industries play a significant role in the Algerian industrial transformation sector, large-scale agribusiness firms dominate the production of major commodities, such as milk, potatoes, wheat, and tomatoes. The tomato sector, in particular, makes a significant national contribution and is coordinated vertically through production contracts to meet domestic demand. A public regulatory mechanism has been in place since 2010 to coordinate the production of processed tomatoes. Understanding the dynamics of tomato production contracts is necessary, especially given the growing domestic demand for processed tomatoes. This understanding highlights public policy concerns and aims to improve intervention efficiency. Consequently, the question arises: How well do these contracts perform? What insights can be gleaned from the observed regularities?

This study aims to empirically analyze the vertical relationship between tomato growers and processors using a comprehensive dataset on contracts from 2018 to 2021. The panel data encompass all contracting tomato growers surveyed over four consecutive years, totaling 9127 tomato growers. This dataset allows for a detailed investigation of contractual performances and their determinants. We examine the proposition that unfair prices lead to lower contractual performances, consequently resulting in low participation in farming contracts. To achieve this, we will first explore and gain insights into the contractual performance levels of the producers. Subsequently, we will analyze the determinants of contractual performance, aiming to identify potential areas for improvement.

The paper is organized as follows: Section 2 provides the conceptual framework of the study. Section 3 outlines the methodology and data. The main results, discussions, and policy implications are presented in Section 4. The final section presents our conclusions.

## 2. Contracting Tomatoes in Uncertain Environment: Concepts and Context

This section explores the main issues within a conceptual framework for this study, focusing first on the fairness assessment in recent theoretical developments and then highlighting the vertical coordination failures in the context of tomato production.

### 2.1. Governance Forms and Contract Farming: Fairness Assessment in Food Supply Chains

The New Institutional Economics framework provides a profound understanding of market and non-market transactions, emphasizing the role of transaction costs. It highlights the importance of contractual aspects in business relationships, where contracts’ emergence and structure are influenced by information asymmetries, incompleteness, moral hazards, and market imperfections [29,30,31,32]. Assessing fairness in exchanges is complex due to the convolutions of human behavior, making sustainable and fair relationships crucial for economic agents of all scales.

Agro-industrial systems are undergoing significant transformation, with contractual relationships becoming essential for global sustainability [32,33]. Modern agri-food chains require fairness to maintain equitable relationships among interconnected actors. Understanding fairness in industrial food systems involves examining three fundamental types of fairness [34]: distributive, procedural, and interactional fairness. Distributive fairness, based on Adams’ equity theory [35], examines the fair distribution of outcomes. In agri-food chains, this involves ensuring fair prices for products and considering perspectives from consumers, producers, and other supply chain partners. Distributive fairness extends beyond pricing to include compensatory justice and establishing fair margins for sellers, highlighting the interconnectedness of equitable revenue distribution within the agri-food chain [36]. Contracts often specify prices and terms in advance, contributing to distributive fairness by ensuring fair compensation for farmers’ produce. Fixed pricing mechanisms protect farmers from market fluctuations, providing predictable income [37,38,39]. Procedural fairness, introduced by Thibaut & Walker [40], focuses on the fairness of processes leading to outcomes within the agri-food chain. This includes negotiation processes, bargaining power dynamics, and decision-making transparency. Fair procedures are characterized by consistency, impartiality, representation, and transparency, shaping the trajectory of fairness within the system [41]. Contract farming enhances procedural fairness by ensuring transparent decision-making, access to resources, and dispute resolution. Clear terms and conditions enhance transparency and ensure mutual understanding of contract terms. Access to resources, such as seeds, fertilizers, and technology, under fair conditions creates a level playing field for farmers. Mechanisms for dispute resolution provide structured processes for resolving disagreements, reducing power imbalances [42,43].

Interactional fairness, proposed by Bies & Moag [44], considers interpersonal treatment during the implementation of procedures within the agri-food chain. This dimension includes both interpersonal and informational fairness, emphasizing respectful, polite, and transparent communication. Quality interactions influence perceptions of fairness and contribute significantly to the overall fairness of the agri-food system [45]. Hence, effective communication builds trust and informs farmers of their rights and responsibilities. Ethical implementation empowers farmers by providing security and stability and creates balanced power dynamics. Fair and ethical treatment, including timely payments and addressing concerns promptly, fosters positive interactions and contributes to the overall fairness of the agro-food system [43,46].

These three types of fairness are interwoven, forming a symbiotic relationship within the agri-food chain. Distributive fairness is achieved through procedural fairness, while interactional fairness enables the effective implementation of fair procedures [45]. This interdependence emphasizes the holistic nature of fairness, reinforcing its significance in fostering a balanced and equitable agri-food system. Enabling fairness within the agri-food chain involves both upstream and downstream practices. Upstream practices address unfair trading practices, ensure transparency, and promote ethical treatment of farmers, while downstream practices focus on cost and price transparency, consumer intentions for fair food, and the perceived value of fairness throughout the food production and distribution cycle [34,47].

On the other hand, contract theory can provide a lens to analyze how contracts can mitigate risks and enhance efficiency within vertical linkages in food supply chains. Contracts serve as tools for managing uncertainty and aligning incentives between different actors in the value chain [15]. In contexts characterized by weak institutional environments, the role of public regulation becomes critical in supporting private contractual arrangements [48]. Public policies can promote contract farming by providing a legal framework that enforces contract compliance and resolves disputes, thereby reducing transaction costs and enhancing trust among stakeholders [17]. Vertical coordination in food supply chains involves the synchronization of production and marketing stages, ensuring the quantity, quality, and timing of product flows. Governance structures, such as formal contracts, play a key role in facilitating this coordination [32]. The presence of robust contractual arrangements can mitigate the risks of market imperfections and ensure that the benefits of value addition are equitably distributed among supply chain participants [49]. Public regulation of private arrangements, including contract farming, can enhance the stability and sustainability of these vertical linkages by addressing power imbalances and ensuring fair practices [50].

The integration of fairness assessments into contract farming practices addresses the unique challenges faced by farmers and processors. Despite varied results from different studies, contract farming, when considering fairness issues, can create a more equitable and sustainable agro-food chain. Correspondingly, the success or failure of vertical coordination among stakeholders within a value chain is necessary for optimal market operation in such contexts.

Findings from various countries highlight the importance of integrating fairness assessments into contract farming practices. In Brazil, findings by Zylbersztajn & Nadalini [19,26] emphasize the critical role of contracts in coordinating between farmers and the processing industry. However, they also point out significant challenges in contract enforcement and frequent opportunistic behavior by farmers. The weak enforcement mechanisms and bureaucratic inefficiencies in Brazil mirror similar issues found in Algeria, resulting in high rates of contract breaches and opportunistic behavior by farmers who take advantage of better market opportunities when contractual prices are unfavorable. Similarly, the Italian tomato sector [51,52] suffers from market concentration where larger firms exert considerable bargaining power over smaller farmers. In Italy, the formation of interbranch organizations and producer organizations aims to balance this power disparity by fostering better coordination among value chain actors. These coordination mechanisms are crucial in mitigating the negative impacts of market concentration, promoting dialogue, and setting reference prices to stabilize market relations.

In Vietnam, contract farming has been shown to significantly improve farmers’ incomes and reduce risks associated with price fluctuations and market access [38,42,53]. However, challenges, such as a lack of transparency and trust between farmers and processors, often undermine the effectiveness of these contracts. Addressing these issues through fairness assessments and transparent contractual arrangements can enhance the sustainability and equity of the food value chain. Moreover, in China, contractual arrangements between farmer cooperatives and buyers have been found to improve market access and income stability for smallholder farmers [50]. Nonetheless, issues of power imbalance and opportunistic behavior remain prevalent. The case of Ghana also highlights the benefits and challenges of contract farming. Arouna et al. [39] and Dubbert [54] found that contract farming could lead to significant improvements in farmers’ incomes and production efficiency. However, the effectiveness of these contracts often depends on the enforcement mechanisms and the level of trust between farmers and processors. The integration of fairness assessments in these diverse contexts underscores the importance of considering distributive, procedural, and interactional fairness in contract farming practices. These assessments can address the challenges faced by farmers and processors, promoting more equitable and sustainable agro-food chains. By learning from international experiences and implementing similar strategies, countries, like Algeria, can improve the stability and efficiency of their agricultural value chains, fostering better relationships between all stakeholders involved.

### 2.2. Vertical Coordination Failure and the Risks of Simplicity

Tomato paste manufacturers must secure the necessary quantity of industrial tomatoes, a crucial input for production, according to production capacity and perceived domestic demand. Managing this process is vital for meeting rising local demand and ensures the efficiency of involved actors, especially given the importance of tomatoes in Mediterranean consumption habits [55,56].

In the context of many developing countries, the industrial tomato is a staple in daily meals, contributing to its significant role in cuisine. Local manufacturers recognize the immense potential of domestic demand for tomato paste, especially double tomato concentrate (DTC). They engage in private arrangements through the spot market with local tomato farmers and import triple tomato concentrate (TTC) to produce DTC. This reliance on imports, coupled with rising demand, has increased costs and created challenges for tomato paste entrepreneurs.

Following the economic reforms of 1989 in Algeria, the industrial tomato sector in many developing countries faced a crisis. Government disengagement and liberal policies led to a surge in TTC imports. While high-yielding varieties of industrial tomatoes were acquired through imports, providing some relief to manufacturers, the heavy reliance on imports posed significant food security concerns. Public intervention, guided by liberal principles, was necessary. In 2010, the government introduced hybrid public–private contractual mechanisms in the form of marketing and production contracts, committing farmers to deliver specified quantities of tomatoes at a fixed price [57,58,59]. Administered by a specialized public office (ONILEV: National Inter-professional Office of Vegetables and Meats), these initiatives facilitated tomato farmers’ participation in contract farming, enhancing efficiency and market stability. Recently, however, concerns have arisen about the potential failure of this vertical coordination mechanism.

Vertical coordination failure in food systems is not unique to Algeria. Around the world, food supply chains face similar issues. In India, the lack of effective vertical coordination in the onion and potato supply chains has led to significant post-harvest losses and price volatility [17]. In Mexico, the failure of vertical coordination in the avocado supply chain has resulted in the uneven distribution of benefits, with smallholder farmers often receiving lower prices due to their weaker bargaining power [60]. These examples highlight the importance of effective vertical coordination in ensuring the sustainability and efficiency of food systems.

The simplicity of contracts in the tomato value chain, which often stipulate only the quantity of tomatoes and a non-negotiable base price, has profound implications on decision-making and income distribution within the sector. These straightforward contract structures are designed to be easy to manage and understand. However, they have led to significant issues for public policy. The lack of price negotiation mechanisms within these contracts can lead to income disparities and financial instability for farmers, which will further exacerbate the rate of contract breaches as farmers seek better opportunities elsewhere.

Simple contract structures, while easier to administer, lead to significant enforcement and compliance issues. In Brazil, the simplicity of contracts has resulted in frequent breaches and disputes, as they do not account for the complexities of agricultural production and market dynamics [19]. Similarly, in Ghana, rigid contract terms have led to compliance issues, as farmers struggle to meet their obligations under varying conditions [39]. In China, the lack of flexibility in contracts has been a barrier to effective enforcement and has resulted in opportunistic behaviors among farmers [50].

## 3. Research Methodology

### 3.1. Data Structure and Sources

This study utilizes a comprehensive dataset on production contracts between processors and tomato growers in Algeria, collected from 2018 to 2021 by a specialized team on a comprehensive list of tomato growers provided by the underlying public office (ONILEV). The processed unbalanced panel data consists of 9127 observations (contracting tomato growers). The data structure and production aggregates are presented in Table 1. The dataset identified the following key variables: contracted area (in hectares), contracted quantity (in tons), actual delivered quantity, and distance from the farm to the processing plant (qualitative variable). Two crucial variables were constructed to reflect contract performance: the Index of Contractual Performance (ICP), representing the percentage of actual delivered quantity relative to the contracted quantity, and a binary variable (BCP), which is 1 if performance exceeded 70% and 0 otherwise. These variables serve as essential indicators for analyzing contract performance.

The dependent variables are ICP and BCP, while the explanatory variables include contracted area (CAR), produced quantity (PROQ), and distance (DIS) from the processing plant. A dummy variable (SD) is constructed, capturing the combined effects of scale and distance from the processor and resulting in four categories: SD1 for small farmers close to the processor, SD2 for small farmers distant from the processor, SD3 for large farmers close to the processor, and SD4 for large farmers distant from the processor. Descriptive statistics are shown in Table 2 and Table 3.

### 3.2. Empirical Modeling Procedure and Hypotheses

Three model specifications have been developed to address the nature of the dependent variable as described by Greene [61] and Baltagi [62]. The first model utilizes a qualitative (binary) dependent variable, employing the logit specification, while the second model employs a censored variable, following the tobit specification, to capture the level of contractual performance. In the first case of estimation, contractual performance (or contract breach) is defined based on whether the production effectively delivered is more than (or less than) 70% of the contracted quantity, represented by the binary variable *BCP*. Field experts determined this threshold, considering expected production losses due to technological and environmental conditions. For the second and third cases of estimation, a censored variable *ICP* is used, ranging from 0 (indicating contract breach) to 1 (representing complete fulfillment of the contract). The quantile regression specification, as described by Koenker et al. [63], is applied in these cases. In all three models, the explanatory variables include farm size, contracted quantity, distance, and the SD dummy variable. Detailed descriptions of the explanatory variables can be found in Table 2 and Table 3. These models aim to shed light on the relationships between these variables and the level of contractual performance.

The logit model specification for the binary dependent variable *BCP* is expressed as follows:

$$logit\left(P\left({BCP}_{i}=1\right)\right)=\beta_{0}+\beta_{i}X_{i}+\epsilon_{i}$$

The tobit model specification for the censored dependent variable *ICP* is expressed as follows:

$${ICP}_{i}^{*}=\beta_{0}+\beta_{i}X_{i}+\epsilon_{i}$$

For this case, the dependent variable *ICP_i_* is defined as follows:

$${ICP}_{i}^{*}=\left\{\begin{array}{ccc} {ICP}_{i}^{*} & if & 0<{ICP}_{i}^{*}<1\\ 0 & if & {ICP}_{i}^{*}\leq 0\\ 1 & if & {ICP}_{i}^{*}\geq 1 \end{array}\right.$$

The quantile regression specification for different quantiles (*τ*) is expressed as follows:

$$Q_{{ICP}_{i}}\left(\tau \right)=\beta_{0}\left(\tau \right)+\beta_{i}\left(\tau \right)X_{i}+\epsilon_{i}\left(\tau \right)$$

where *X_i_* represents the set of the four explanatory variables, and *τ* = {0.05, 0.25, 0.50, 0.75, 0.95} represents the quantiles. Based on theory and previous studies, the following hypotheses were formulated:

**Hypothesis** **1.** *The level of contractual performance is positively related to the scale of the farm.* Larger farms are more likely to engage in specific investments due to their greater resource base. They tend to be more sensitive to greater losses because a significant portion of their income depends on the volume of produce delivered under contract. This dependence incentivizes larger farms to maintain long-term relationships with the processing plant, ensuring stable and predictable income streams. Economies of scale also play a crucial role, as larger farms can reduce production costs per unit of output, thus increasing their competitiveness and ability to meet contractual obligations. Additionally, larger farms often possess stronger bargaining power, enabling them to negotiate more favorable contract terms, which further enhances their ability to perform well under contract farming arrangements. This hypothesis is supported by the transaction cost economics framework, which posits that larger entities can better absorb transaction costs and uncertainties, thereby improving contractual performance [64,65].

**Hypothesis** **2.** *There exists a negative relationship between the level of contractual performance and the contracted quantity.* Farmers with larger contracted quantities face higher production risks, making it challenging to meet the agreed-upon quantities consistently. These risks include variability in weather conditions, pest infestations, and other unforeseen agricultural challenges that can significantly impact yield. Larger contracts imply higher stakes for both farmers and processors, as the failure to deliver the agreed-upon quantity can lead to substantial financial losses and strained relationships. The associated risks may lead to a lower likelihood of contractual performance as the contracted quantity increases, as farmers may struggle to manage the complexities and uncertainties involved in meeting large-scale production demands. This hypothesis aligns with the spatial economics theory, which suggests that distance affects transaction costs and market accessibility, thereby influencing economic behavior [26,37,66,67,68,69].

**Hypothesis** **3.** *The level of contractual performance is influenced by the distance between farms and the contracting processor.* Proximity to processors can provide farmers with easier access to alternative markets and better price information, potentially leading to contract breaches when local market prices are more favorable. Conversely, farmers located at greater distances may have limited market access and become more reliant on the processor, enhancing their commitment to contract compliance. This dependency motivates them to honor contracts, as breaching could jeopardize their primary market outlet. Studies by Bellemare et al. [14], Simmons et al. [68], and Arouna et al. [39] support this dynamic, highlighting how distance affects transaction costs, market behavior, and contractual compliance. Additionally, Jia & Huang [50] and Zylbersztajn & Nadalini [19,26] demonstrate that distant farmers’ investment in efficient logistics and reliance on contracts contribute to higher compliance rates, underscoring the complex relationship between distance and contractual performance.

Before conducting the econometric modeling, visualization tools will be used to explore the contractual performance index. This step is essential to gain a broad understanding of farmer performances in the value chain in this context. The visualization tools we will employ include dynamic histograms of frequency distribution and the Gaussian kernel density estimation in dynamic pooled plots. These will help us to obtain an overview of the distribution of contractual performances and to identify any potential patterns or trends that could influence the analysis.

## 4. Results and Discussion

**Figure 1:**
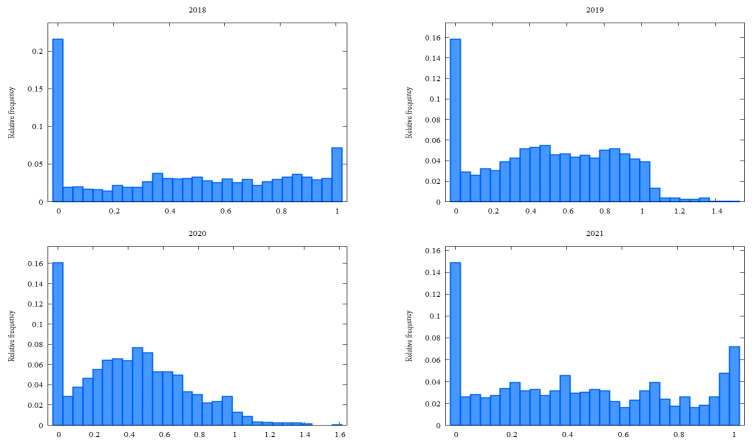
Dynamic histograms for the frequency distributions of the contractual performances of tomato production.

This section initially focuses on the dynamic exploration of contractual performance within the vertical relationship under investigation. To illustrate the distribution of contractual performance, a frequency diagram has been created, as depicted in Figure 1. This figure shows the frequency distribution of contractual performance as a percentage of the contracted quantity delivered.

From Figure 1, it becomes evident that contractual defaults play a dominant role in the behavior of contracting producers. On average, over the four consecutive years, poor contractual performance (i.e., a commitment rate close to zero) accounts for almost 18% of the cases, while ideal performance (i.e., a commitment rate close to 100%) constitutes approximately less than 5% on average. Additionally, it is crucial to highlight the significant presence of intermediate commitment rates. These findings indicate a diverse range of performance levels among the contracting farmers.

The significant presence of contractual defaults, representing nearly 18% of the cases on average, highlights a concerning issue within the tomato sector. Such defaults indicate instances where the contracted quantity of tomatoes is not effectively delivered, leading to breaches in the agreed-upon contracts. On the other end of the spectrum, ideal contractual performance, where the contracted quantity is fully delivered as per the agreement, constitutes around 5% on average. This indicates that a small fraction of contracting farmers consistently adhere to their contractual commitments, delivering the contracted quantity with high precision. Interestingly, a substantial portion of the observed contractual performance lies in the intermediate commitment rates. This implies that a considerable number of farmers deliver a portion of the contracted quantity but fall short of fulfilling the entire commitment.

**Figure 2:**
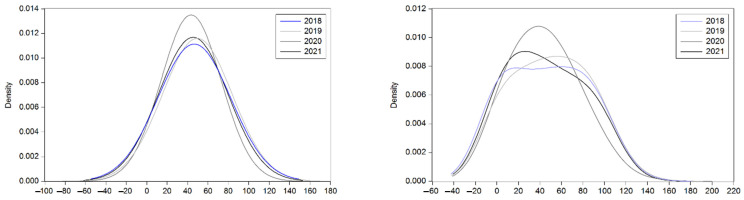
Theoretical and empirical kernel densities for contractual performances in tomato production.

To gain an ample visualization of the dynamics of contractual performance, it is more informative to analyze density functions. The theoretical density function serves as a basis, followed by empirical adjustments based on actual data, as depicted in Figure 2. The lefthand side of the figure shows the theoretical densities for each of the four years, while the righthand side displays the corresponding empirical densities. Upon observing both illustrations, we can infer that, theoretically, contractual performance tends to exhibit a stable behavior, centered around a mediocre level of performance, approximately 50%. However, when examining the empirical evidence, some interesting patterns emerge. The initial observation in 2018 reveals a slight bimodal distribution, indicating that a significant portion of contracting producers exhibited extreme behavior in terms of contractual commitment. On one end of the spectrum, some farmers struggled to meet their contractual obligations, resulting in weak performance. On the other end, some farmers excelled in contractual fulfillment, achieving an ideal level of performance. As the analysis progresses for the subsequent two years, a shift is observed in the empirical density function. The distribution becomes unimodal, centered around low performance. However, in the most recent year (2021), a return to bimodality occurs in the empirical density function, with a notable shift towards mediocre performance. This suggests that some farmers have managed to improve their contractual commitment, moving from the poor performance category to a more acceptable level. On the other hand, weak performance persists among producers, indicating that challenges in meeting contractual obligations persist.

To discern the key determinants of contractual performance among contracting producers, the findings from the logit, tobit, and quantile regressions are presented in Table 4. The quantile regression analysis explores five categories of contractual performance, namely poor, weak, median, regular, and perfect performances (for 0.05, 0.25, 0.50, 0.75, and 0.95 quantiles, respectively), allowing for an examination of a variety of performance levels. The estimations from all the model specifications exhibit a high level of statistical significance, as indicated by the likelihood ratios and F-test statistics for overall significance. This robust significance level ensures the reliability and suitability of the models for straightforward interpretation of the results.

Firstly, the logit and tobit estimations show that farm size has a significant positive effect on contractual performance. Larger farms are more likely to exhibit better contractual performance, which aligns with the hypothesis. Larger farms have greater resources, capacity for investment, and financial stability, making them more capable of fulfilling their contractual obligations. Moreover, these farms may have developed long-term relationships with processing plants, and a significant portion of their income might depend on the success of these contracts. Hence, they are motivated to honor the agreements to secure their livelihood. The quantile regression results further confirm the positive effect of farm size but indicate that the significant impact is primarily observed above the median performance level. For lower levels of contractual performance, farm size may not be a dominant factor. This could be because smaller farms are more vulnerable to price risks and are less likely to invest in long-term relationships with processors, which might lead to lower commitment levels. This is in line with the results of Guo & Jolly [23] and corroborates the results of Zylbersztajn & Nadalini [19,26].

Moreover, the logit and tobit estimations consistently show that contracted quantity has a significant negative effect on contractual performance. This result is in line with the hypothesis. The negative relationship suggests that as the contracted quantity increases, the likelihood of contractual performance decreases. It implies that farmers with larger contracted quantities are more prone to contract breach or underperformance. One plausible explanation for this negative relationship is that larger contracted quantities lead to higher production risks for farmers. They face challenges in meeting the exact quantity demanded by the processor due to uncertainties in weather conditions, pest infestations, or other factors beyond their control. Consequently, farmers might be reluctant to agree to larger quantities to minimize the risk of non-compliance. The quantile regression results also confirm the negative impact of contracted quantity, which remains significant at the 1% level above the regular performance level. This suggests that even at higher performance levels, the contracted quantity still has a significant negative effect, implying that farmers with larger contracted quantities may find it challenging to consistently meet the agreed-upon quantity. This is corroborated by the results of Zylbersztajn & Nadalini [19,26].

The simultaneous effect of farm size and contracted quantity on contractual performance reveals nuanced dynamics that align with our hypotheses. The positive impact of farm size on contractual fulfillment supports the hypothesis that larger farms are more likely to meet their contractual obligations due to their greater resources, investment capacities, and efficiency in meeting processing plants’ demands. Larger farms benefit from economies of scale and stronger bargaining power, enabling them to negotiate more favorable contract terms and deliver larger quantities of tomatoes as agreed. Conversely, the negative impact of contracted quantity on contractual performance, as observed, indicates that as the contracted quantity increases, the likelihood of contractual fulfillment decreases (as corroborated by Gulati et al. [17]; Simmons et al. [68]; Zylbersztajn & Nadalini [26]). This suggests that larger contracted quantities pose higher production risks and management challenges, especially for smaller farms with limited resources. Smaller farms may struggle to meet larger contract volumes, resulting in lower contractual performance. This finding highlights the importance of considering the specific capacities and constraints of individual farms when designing and negotiating contracts. A tailored approach to contracting, which takes into account the varying capabilities of farms, is essential for improving overall contractual performance and ensuring sustainable relationships within the tomato sector.

Additionally, the logit estimation does not show a statistically significant effect of distance, while the tobit estimation reveals a statistically significant negative effect at the 1% level. This finding does not support the hypothesis, which suggests a strong relationship between contractual performance and distance. However, it is essential to consider that the tobit model accounts for censored data and captures both the likelihood of performance (non-zero values) and the extent of performance (actual performance values). The quantile regression results indicate that the effect of distance is evident with high significance levels (at least 5%) above the weak performance level. This effect is negative for weak performances, but it becomes positive beyond this level. This result is explained by the fact that farmers located closer to the contracting processor have better access to market channels, price information, and processing plant requirements. As a result, they are more aware of potential risks and opportunities in the market, leading to better performance. On the other hand, farmers located farther away from the processor have limited access to market information, leading to more uncertainty and risk in the contractual arrangement. However, once farmers achieve a certain level of performance (above the weak performance level), the positive effect of distance becomes evident, because these farmers have developed efficient transportation systems, established stable relationships, or have built expertise in managing long-distance contracts.

The analysis of the simultaneous effects of farm size and distance, using the SD1 to SD4 categories, reveals interesting findings. The first three categories (SD1, SD2, and SD3) generally do not show statistical significance in most cases, while the fourth category (SD4) demonstrates high positive significance (at the 1% level) with the exception of the poor level of contractual performance in the quantile regression. This implies that large-scale farms located far from the contracting processor positively influence contractual performance, as they are more likely to honor their contracts. The combination of scale and distance appears to have a significant impact on performance, but only for large farms located at a considerable distance. These findings suggest that large farms at a significant distance from the processor have developed efficient logistics, transportation, and communication systems, enabling them to overcome the challenges associated with long-distance contracts.

These results are consistent with findings from other regions, where similar dynamics have been observed. For instance, in Vietnam, research on contract farming for high-value crops, such as coffee and cocoa, has shown that larger farms with better access to resources and infrastructure tend to exhibit higher contractual performance [42]. Similarly, in Ghana, the integration of smallholder farmers into the supply chains of multinational companies has highlighted the importance of scale and logistical efficiency in improving contract compliance and overall performance [39].

The contrasting effects of farm size and contracted quantity on contractual performance highlight the need for tailored policy interventions. Large farms benefit from economies of scale and better access to resources, which enhances their contractual performance [11]. However, increasing the contracted quantity alone may not yield better performance, especially for smaller farms [37]. This underscores the importance of considering the unique capacities and constraints of different farm sizes when designing contract farming schemes.

Moreover, the mixed findings regarding the effect of distance suggest that proximity to processors does not uniformly enhance contractual performance. While closer proximity might provide better market information and opportunities for side-selling, it also introduces risks of contract breaches if local market conditions are more favorable [7]. In contrast, distant farms might develop stronger relationships with processors due to their dependence on the contractual arrangement, although they also face logistical challenges that can impact performance [10]. These mixed findings indicate that distance alone cannot predict contractual performance.

The analysis of contractual performance within the tomato processing supply chain reveals several key policy implications to improve the situation and incentivize tomato growers to fulfill their contracts. Ensuring fair price setting is paramount for enhancing distributive fairness. To improve contractual performance, processors need to set fair and reasonable prices for tomato production. Prices should be based on market conditions, annually actualized production costs, and a fair profit margin for both parties. Transparency in price determination and regular reviews of prices in accordance with market fluctuations will help build trust between processors and tomato growers. This mirrors the efforts in Italy, where interbranch organizations and producer organizations set reference prices to stabilize market relations by fostering better coordination among value chain actors [51,52]. Implementing similar mechanisms in this case could enhance price fairness and contractual compliance.

Enhancing communication and trust in the grower–processor relationship is manifestly important for improving procedural and interactional fairness. Establishing effective communication channels between processors and tomato growers will foster trust and mutual understanding. Regular consultations and discussions on contract terms, prices, and other concerns can alleviate the perception of unfairness and strengthen relationships. In Brazil, Zylbersztajn & Nadalini [19,26] emphasize the importance of transparent and open communication to mitigate opportunistic behavior and improve contract adherence. Furthermore, ensuring transparent decision-making processes will address procedural fairness by involving all stakeholders in contract negotiations and adjustments.

Processors can offer various incentives to encourage tomato growers to fulfill their contracts, enhancing both distributive and interactional fairness. These incentives could include bonuses for meeting or exceeding contract targets, providing technical support or training to improve production efficiency, and offering long-term contracts to provide stability and security for growers. From the side of public policy involvement in the coordination process, the public office should intervene by readjusting current support and subsidies to the tomato industry, particularly to small-scale growers. These strategies have been observed in Italy and Brazil, where government support plays a significant role in stabilizing the agricultural sectors [26,51].

Introducing flexibility in contracts also helps to mitigate risks for both processors and tomato growers, contributing to procedural fairness. Allowing adjustments in contracted quantities based on unforeseen circumstances, such as weather- or price-related issues, will provide more security for growers and prevent contractual breaches due to uncontrollable factors. This approach aligns with findings from international contexts, where adaptive contracting strategies have been shown to improve contractual performance and farmer satisfaction [37].

Further attention should be given to enforcement and dispute-resolution mechanisms, which are decisive for maintaining procedural fairness. Establishing efficient enforcement mechanisms and dispute-resolution procedures can assure both parties that contractual obligations will be met. This can involve a third-party arbitrator to resolve disputes and enforce the terms of the contract if necessary. The Italian experience with producer organizations demonstrates the effectiveness of such mechanisms in promoting dialogue and setting reference prices to stabilize market relations [52].

To leverage growers’ bargaining power, contracting farmers could adopt a market diversification strategy for other crops and productive activities, enhancing distributive fairness. Encouraging diversification in the market by exploring opportunities for tomato exports or value-added products can reduce dependency on a single contracting processor. This will strengthen the bargaining power of tomato growers and give them more leverage in contract negotiations. In Brazil, diversification has been key to providing farmers with alternative income sources, thereby reducing their dependency on single-crop contracts [26].

The issues discussed in this study are mirrored in the context of the tomato sector in Brazil and Italy, as highlighted by various other studies. In Brazil, Zylbersztajn & Nadalini [19,26] emphasize the critical role of contracts in coordinating between farmers and the processing industry, yet point out significant challenges in contract enforcement and frequent opportunistic behavior by farmers. Both the Brazilian and Algerian contexts suffer from weak public enforcement mechanisms and bureaucratic inefficiencies that undermine contractual stability, resulting in high rates of contract breaches. Farmers often take advantage of better market opportunities when contractual prices are unfavorable, leading industries in both countries to consider relocating or restructuring their contractual approaches due to these persistent enforcement issues. Similarly, the Italian tomato sector, as studied by Bertazzoli et al. [51] and Čechura et al. [52], suffers from market concentration where larger firms exert considerable bargaining power over smaller farmers. In Italy, the formation of interbranch organizations and producer organizations aims to balance this power disparity by fostering better coordination among value chain actors. This approach parallels efforts in Algeria, where forming cooperatives will enhance farmers’ negotiating power and ensure fairer pricing and value distribution. These coordination mechanisms in both regions are critical in mitigating the negative impacts of market concentration, promoting dialogue, and setting reference prices to stabilize market relations. Encouraging similar initiatives in Algeria could potentially lead to more equitable and stable market conditions.

Finally, addressing the issue of unfair price perception by processors and incentivizing tomato growers to fulfill their contracts requires a collaborative effort involving all stakeholders, including processors, tomato growers, and public policymakers. By implementing fair and transparent pricing mechanisms, enhancing communication and trust, and providing appropriate incentives and support, the industrial tomato sector in Algeria can achieve higher contractual performance, contributing to its overall growth and sustainability. This collaborative approach mirrors successful strategies mainly in Italy and Brazil, where multi-stakeholder initiatives have led to more equitable and stable market conditions [19,51].

## 5. Conclusions

The contracting dynamics in the tomato sector hold immense significance not only for Algeria but also for any developing country. As a crucial component of the food value chain, industrial tomato production and processing play a vital role in meeting the growing demand for this staple food item. The findings of this study have shed light on the complex relationships between tomato growers and processors, uncovering the factors that influence contractual performances.

This study aimed to empirically analyze the tomato grower–processor vertical relationship in Algeria through a comprehensive dataset on contracts from 2018 to 2021, encompassing 9127 tomato growers. The detailed investigation allowed for an in-depth examination of contractual performances and their determinants. Using logit, tobit, and quantile regressions, the study identified key factors influencing contractual performance among contracting producers. Farm size, contracted quantity, and distance from the contracting processor emerged as significant determinants of contractual behaviors. The simultaneous effects of scale and distance revealed nuanced patterns of influence on contractual performance at different levels. These findings provided insights for policymakers and industry stakeholders to design targeted interventions and improve the efficiency and stability of vertical coordination mechanisms within the sector.

Based on these findings, several recommendations have been made to incentivize tomato growers and improve overall contractual performance. Processors should adopt a fair and transparent pricing mechanism to ensure equitable compensation for tomato production. Effective communication channels between stakeholders should be established to facilitate negotiations and build trust. Processors can provide incentives and support to tomato growers, such as bonuses, technical assistance, and long-term contracts, to enhance commitment. Public policymakers should also implement more supportive policies that promote access to credit, modern farming technologies, and infrastructure improvements for the tomato industry. Additionally, flexibility in contracts, efficient enforcement mechanisms, and dispute-resolution procedures are essential to mitigate risks and ensure contractual compliance. By adopting these strategies, the industrial tomato sector can foster better relationships between processors and growers, leading to improved contractual performance and overall growth.

While the above analysis provides understandings of the determinants of contractual performance in the tomato sector, there are some limitations and potential avenues for further exploration. The analysis primarily focused on internal factors within the contractual relationship. However, external factors, such as market fluctuations, climate change, and changes in government policies, can also influence contractual performance. Future studies should consider incorporating these external factors to develop a more comprehensive understanding of the determinants of contractual performance. Moreover, the study assumed linearity in the relationship between contract performance and farm size and distance. Contractual performance may exhibit non-linear responses to these factors. Investigating the existence of non-linear relationships and the potential benefits of flexible contracts could provide more insights for improving the efficiency and stability of the tomato sector.

Additionally, the study focused on short-term contractual performance; however, the long-term impact of contract farming on tomato growers’ livelihoods, as well as on the sustainability of the sector, should be explored to gauge the overall effectiveness of contract farming arrangements. Incorporating these considerations and further exploring the above aspects would contribute to a more comprehensive and robust understanding of the dynamics and challenges of the tomato sector in developing countries. This broader perspective could potentially inform policy interventions and industry practices for sustainable growth.

## Figures and Tables

**Table 1:** The data structure of the unbalanced panel of tomato production contracts.

Year	2018	2019	2020	2021	Total
Number of contracting tomato growers	3713	2627	1867	920	9127
Delivered quantity (in 1000 tons)	642	499	538	351	2030

**Table 2:** Variables definition and their overall descriptive statistics.

Variable	Symbol	Definition	Obs.	Mean	Median	S.D.	Min.	Max.
Index of Contractual Performance	ICP	Censored variable (from 0 to 1)	9172	0.456	0.452	0.33	0	1
Binary Contractual Performance	BCP	Binary variable (0 and 1)	9172	0.284	0.000	0.45	0	1
Contracted area	CAR	Quantitative variable	9172	5.480	4.000	5.11	0.6	85.0
Contracted quantity	PROQ	Quantitative variable	9172	44.800	30.000	56.40	3.0	875.0
Distance	DIST	Qualitative variable (from 1 to 4)	9172	0.968	1.000	1.02	0	4
Scale and Distance	SD	Dummy variable						
		SD1	5031	0.549	1.000	0.498	0	1
		SD2	1569	0.171	0.000	0.377	0	1
		SD3	1889	0.206	0.000	0.404	0	1
		SD4	683	0.074	0.000	0.263	0	1

**Table 3:** The data structure of the unbalanced panel of tomato production contracts.

Variables	Obs.	Mean	Median	S.D.
ICP 2018	3758	0.461	0.464	0.350
ICP 2019	2627	0.473	0.476	0.344
ICP 2020	1867	0.459	0.458	0.334
ICP 2021	920	0.456	0.452	0.335
BCP2018	3758	0.314	0.000	0.464
BCP2019	2627	0.319	0.000	0.466
BCP2020	1867	0.288	0.000	0.453
BCP2021	920	0.284	0.000	0.451
CAR 2018	3758	5.165	4.000	4.465
CAR 2019	2627	5.251	4.000	4.705
CAR 2020	1867	5.521	4.000	5.213
CAR 2021	920	5.478	4.000	5.110
PROQ 2018	3758	36.055	30.000	37.446
PROQ 2019	2627	37.185	30.000	40.869
PROQ 2020	1867	41.418	30.000	48.925
PROQ 2021	920	44.805	30.000	56.412
DIST 2018	3758	1.074	1.000	1.071
DIST 2019	2627	1.019	1.000	1.069
DIST 2020	1867	0.951	1.000	1.053
DIST 2021	920	0.968	1.000	1.024

**Table 4:** Results of the quantile regression estimation for the contractual performance.

Variables	Logit Estimates	Tobit Estimates	Quantile Regression Estimates				
	LR(6): 35.2 ***	LL: 422.1 ***	Poor	Weak	Median	Regular	Perfect
	R: 0.003	F: 3.17 ***	0.05	0.25	0.50	0.75	0.95
Farm size	0.018 ***	0.002 **	−0.001	−0.001	0.002 ***	0.001 *	0.008 ***
	(3.32)	(2.25)	(−0.000)	(−0.47)	(2.38)	(1.11)	(13.1)
Quantity	−0.002 ***	−0.001 **	0.001	0.002 *	−0.001	−0.003 ***	−0.001 ***
	(−4.33)	(−1.99)	(0.000)	(1.26)	(−1.56)	(−2.65)	(−18.2)
Distance	0.030	−0.012 ***	−0.001	−0.037 ***	0.006 **	0.009 **	0.008 ***
	(1.33)	(−2.92)	(−0.000)	(−4.28)	(1.32)	(1.42)	(32.3)
SD1	−0.094	−0.027	−0.001	−0.039 *	−0.009	−0.009	−0.001
	(−1.04)	(−1.59)	(−0.000)	(−1.12)	(−0.50)	(−0.37)	(−0.000)
SD2	−0.145	−0.023	−0.001	−0.005	−0.009	−0.025	−0.003 ***
	(−1.43)	(−1.21)	(−0.000)	(−0.143)	(−0.41)	(−0.86)	(−2.71)
SD3	0.079	0.014	0.001	0.032	0.014	0.029	0.001
	(0.81)	(0.75)	(0.000)	(0.83)	(0.69)	(1.02)	(0.97)
SD4	−0.885 ***	0.450 ***	−0.001	0.169 ***	0.443 ***	0.746 ***	0.999 ***
	(−9.76)	(25.5)	(−0.000)	(4.742)	(22.2)	(28.0)	(898)

Note: asterisks for significance level: *** for 1%, ** for 5%, and * for 10%.

## Data Availability

The original contributions presented in the study are included in the article, further inquiries can be directed to the corresponding author.
